# A social science trust taxonomy with emergent vectors and symmetry

**DOI:** 10.3389/fpsyg.2024.1335020

**Published:** 2024-08-30

**Authors:** Anthony E. D. Mobbs, Simon Boag

**Affiliations:** School of Psychological Sciences, Faculty of Medicine, Health and Human Sciences, Macquarie University, Sydney, NSW, Australia

**Keywords:** trust, dominance, cooperation, metrology, vectors, symmetry, dimensional models, lexical analysis

## Abstract

**Introduction:**

Trust is foundational to all social science domains, but to date, there is no unifying theory or consistent measurement basis spanning the social sciences. This research hypothesized that trust forms the basis of an ontology that could unify the social science domains. The proposed ontology comprises a Cartesian plane with axes self-trust and other-trust. Self-trust manifests in dominant behaviors, and other-trust manifests in cooperative behaviors. Both axes are divided into five discrete categories, creating a matrix of 25 cells. All words in the lexicon are allocated into one of these 25 cells.

**Methods:**

This research started with an existing 14,000-word lexicon of dominance and affiliation. The lexicon was extended by manually identifying and including socially descriptive words with information regarding self-trust, other-trust, dominance, and cooperation. The taxonomy was optimized using the Gradient Descent machine learning algorithm and commercially curated synonyms and antonyms. The t-test was employed as the objective (or loss) function for Gradient Descent optimization. Word vectors were identified using groups of four words related as synonyms and antonyms.

**Results:**

Over 30,000 words were identified and included in the lexicon. The optimization process yielded a t-score of over 1,000. Over 226,000 vectors were identified, such as malevolent-mean-gentle-benevolent. A new form of symmetry was identified between adjectives and verbs with a common root; for example, the words *reject* and *rejected* are horizontally reflected.

**Discussion:**

The word vectors can create a metrologically compliant basis for psychometric testing. The symmetries provide insight into causes (verbs) and effects (adjectives) in social interactions. These vectors and symmetries offer the social sciences a basis of commonality with natural sciences, enabling unprecedented accuracy and precision in social science measurement.

## 1 Introduction

The social sciences seek to understand human behavior and interactions (Drost, 2011). However, social science domains, such as anthropology, economics, psychology, and sociology, lack unifying theories and are largely isolated from each other, each with its own vocabulary and theoretical constructs (Hartmann, 1939; Lynn and Vanhanen, 2012; Green, 2015; Buyalskaya et al., 2021). Unifying concepts like intelligence have been proposed (Lynn and Vanhanen, 2012). However, while intelligence strongly correlates with educational and vocational outcomes (Ritchie and Tucker-Drob, 2018), intelligence weakly correlates with other social science constructs, such as personality (Anglim et al., 2022). Therefore, although intelligence may conceptually unite some aspects of social science, intelligence cannot be claimed to unify all social sciences.

Conversely, the natural sciences are unified by two theories: quantum mechanics and general relativity (Fröwis et al., 2018; Howl et al., 2019). A benefit of unification is that these theories describe all observable reality, and all other natural science theories are derivable from them. This research sought to identify an ontology that similarly unifies the social sciences, just as quantum mechanics and general relativity unify the natural sciences.

### 1.1 Lexical hypothesis

The lexical hypothesis states that “… all aspects of human personality which are or have been of importance, interest, or utility have already become recorded in the substance of language” (Cattell, 1943) or alternatively that “… people encode in their everyday languages all those differences between individuals that they perceive to be salient and that they consider to be socially relevant in their everyday lives” (Uher, 2013). A corollary of the lexical hypothesis is that words may be used to identify the structure, or dimensionality, of the concepts being communicated (Uher, 2013). From this corollary, the lexical hypothesis has been extensively used in psychology to infer the characteristics and dimensionality of personality (Cutler and Condon, 2022). Such analysis started with Galton (1884), who introduced a thesaurus to identify a lexicon of more than 1,000 personality-related words. Allport and Odbert extended this approach to identify a lexicon of 18,000 words that describe personality (Allport and Odbert, 1936). Current lexical research typically involves vast empirical studies in which many individuals describe themselves according to questions that assess various personality traits. These questions usually require a Likert-type response (Wirth and Edwards, 2007). Various five and six-factor models have been derived from such research (Goldberg, 1990; Lee and Ashton, 2004; McCrae and Costa, 2008).

### 1.2 Ontology-lexicon-taxonomy approach

Historically, factorization has been the preferred method for using the lexical hypothesis to investigate concepts such as personality (Cutler and Condon, 2022). One problem with this approach is that factor-derived dimensions are sometimes criticized as atheoretical (Eronen and Bringmann, 2021) and reified (Boag, 2015). As an alternative, the current study proposed a three-step analytical approach widely used in the information sciences (Medelyan et al., 2013). This three-step process comprises hypothesizing a candidate ontology, lexicon curation, and taxonomization (Smith, 2012; Nickerson et al., 2013). *Ontologies* specify a domain’s scope and a method for classifying phenomena. *Lexicons* are curated vocabularies describing phenomena within the scope of the ontology. *Taxonomies* arrange the lexicalized phenomena congruent with the ontology.

For example, consider the science of cartology. Cartology’s ontology specifies two perpendicular axes, North–South and East–West, pragmatically divided into 360 equidistant lines of latitude and longitude. This ontology is often represented as a compass and could be called a compass ontology. The lexicon of cartology includes place names such as London, Beijing, New York, and Sydney. The taxonomy of cartology specifies the latitude and longitude as Cartesian coordinates of each place name listed in the lexicon; for example, the location of the Sydney Opera House is precisely specified according to the compass ontology using the coordinates (33.8568°S, 151.2153°E).

A second example is computer graphics color, for which the Red-Green-Blue (RGB) ontology is ubiquitous (Faridul et al., 2016). The RGB ontology specifies three orthogonal dimensions, with each axis pragmatically divided into 256 equidistant divisions. This ontology uniquely specifies 16 million colors, thus creating the RGB taxonomy. The lexicon of colors includes words such as orange, yellow, black, and white. The RGB taxonomy precisely specifies *yellow* with the tuple (255,255,204).

The first step in theory development using the ontology-lexicon-taxonomy approach is to propose a candidate ontology as a working hypothesis. An ontology universally relevant to the social sciences requires identifying a concept foundational to all social science domains. Trust is a candidate concept as it has been widely reported as being significant to many social sciences (Hosking, 2014; Dunning et al., 2019; Krueger and Meyer-Lindenberg, 2019; Weiss et al., 2021). Social science domains reporting trust as a foundation include government (Citrin and Stoker, 2018), politics (Newton et al., 2017), law (Trust, Distrust and the Rule of Law, 2020), economics (Fehr, 2009), sociology (Robbins, 2016), public health (Bargain and Aminjonov, 2020), anthropology (Johnson and Cullen, 2017), commerce (Sarkar et al., 2020), religion (Hall et al., 2015), music (Quinney, 2023), psychology (Campbell and Stanton, 2019; Weiss et al., 2021), and game theory (Han et al., 2021). *Trust* has been defined as a positive expectancy that results in the willingness to cooperate (Kirton, 2020). Empirical research has established high correlations between trust and cooperation (Balliet and Van Lange, 2013). Trust is sometimes thought of as purely an interpersonal construct (other-trust), yet others suggest trust is a two-dimensional concept where trust in oneself (self-trust) is equally meaningful (Ainsworth and Bowlby, 1991; Parkes, 2011, 2013; Dormandy, 2020; Foley, 2020). Alternative descriptions such as agency, self-confidence, and self-efficacy have often been used for the self-trust dimension (Bandura, 1982; Abele and Wojciszke, 2018). Therefore, the present research hypothesized self-trust/dominance and other-trust/cooperation as the axes of its candidate ontology. The dual representation of the latent and manifest in the definition of each axis is necessary to ensure that the development of the lexicon captures the full range of relevant words to test the research hypothesis.

The second step using the ontology-lexicon-taxonomy method is to develop a lexicon relevant to the candidate ontology. Ideally, lexicons should be comprehensive and extensible (Nickerson et al., 2013). In this context, comprehensiveness requires an exhaustive curation process, while extensibility requires that new words be easily added to the lexicon as identified. Several lexicons relevant to personality psychology have previously been curated (Allport and Odbert, 1936; Norman, 1967; Goldberg, 1993; Mobbs, 2020). These lexicons range in size from 600 to 20,000 words. To our knowledge, no comprehensive lexicon combining self-trust, other-trust, dominance, and cooperation has yet been curated. This research proposed to expand upon the largest of these existing lexicons, the Atlas of Personality, Emotion, and Behavior (Atlas) (Mobbs, 2020), to create a comprehensive lexicon. To distinguish the Atlas and Trust lexicons, The *Atlas lexicon* will be described in full, whereas the *Trust lexicon* may be described simply as the *lexicon*.

The final step is taxonomization, where words in the lexicon are classified according to the candidate ontology (Nickerson et al., 2013). A taxonomy’s efficacy is measurable by the degree to which similar phenomena (synonyms) are tightly clustered while dissimilar phenomena (antonyms) are widely separated (Bailey, 1994). The measurability of taxonomic efficacy allows multiple candidate ontologies to be compared. The candidate ontology with the highest taxonomic efficacy is preferred.

Historically, domain experts have manually performed taxonomic classification. For example, cartography was assisted with a compass and sextant before the development of GPS. Automation techniques like machine learning have recently assisted or replaced manual classification (Nguyen et al., 2019; Raghavendra et al., 2019). Gradient Descent is a common approach to machine learning where an objective function is identified that quantifies a desirable taxonomic quality, such as efficacy (Ray, 2019; Gambella et al., 2021). The objective function is then calculated for a multitude of taxonomic variants. The variant maximizing the objective function is considered optimal (Koziel and Yang, 2011). In the context of this research, the objective function is a quantitative measure of the extent to which similar phenomena (synonyms) are tightly clustered and, concurrently, dissimilar phenomena (antonyms) are widely separated (Bailey, 1994). A suitable quantitative measure is the Student’s *t*-test (Verma et al., 2020; Livingston, 2004) using commercially curated synonyms and antonyms (Merriam-Webster, 2021; Oxford University Press, 2022).

Ideal taxonomies are both efficacious and realistic. The choice of trust as the basis of the ontology increases the sense of realism due to the significance of trust across social science. Other features of realistic taxonomies are geometric features, such as symmetries and vectors. Symmetries are ubiquitous throughout natural science (Anderson, 1972; Rattigan et al., 2023). For example, in biology, symmetry is observed as bilateralism (Cardini, 2016), bipedalism (Mobbs et al., 2022), homeostasis (Yang et al., 2015), heartbeats (Odinaka et al., 2010), linguistics (Miestamo, 2007), and neural activity (Tozzi and Peters, 2016). Antonyms also exhibit geometric symmetry, with antonyms considered geometric opposites and equidistant from an origin lying on a vector (Trillas and Alsina, 2007; Li, 2017; Rizkallah et al., 2023). English contains several vectorial representations of natural phenomena, such as freezing-cold-hot-boiling for temperature. This research sought to find novel symmetries and word vectors to align the social sciences with the natural sciences.

### 1.3 Vectors

Scientific variables are expressed as scalar or vector quantities (Joshi and Kumar, 2004). Scalar quantities offer no information on the directionality to which the quantity is applied. Conversely, vectors express both a quantity and direction. For example, a vehicle traveling at 60 kilometers per hour is a scalar quantity because it offers no information about the direction of travel. By adding information about the direction of travel, for example, North, the variable becomes a vector. Therefore, a vehicle traveling 60 kilometers per hour North has been expressed as a vector quantity.

**Figure 1:**
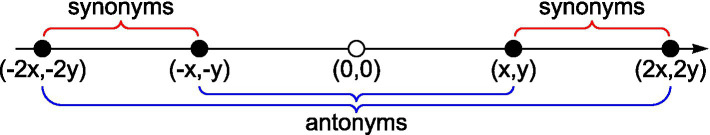
Symmetric word vectors. Word vectors are defined as four words on a line starting and ending at the outer edges of the trust ontology and passing through the origin. The outer- and inner pairs of words are associated with each other as antonyms. The left and right pairs are associated with each other as synonyms. The distance between the synonyms can be calculated using the Pythagorean theorem. For example, when the vector lies on the horizontal axis (y = 0), the distance between synonyms is 1, and the distance between antonyms is 4 for the outer pair of antonyms and 2 for the inner pair.

**Figure 2:**
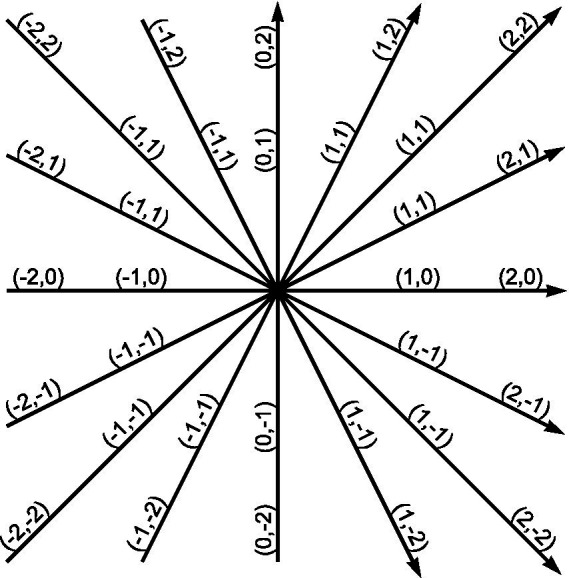
Cardinal vectors spanning the trust taxonomy. The Cartesian coordinates of points along each vector are shown. The Cartesian coordinates also form a 5×5 matrix with 25 cells.

Vectors are often represented on Cartesian coordinate systems, comprising measured orthogonal axes. Ontologies described as Cartesian coordinate systems, such as the compass ontology, facilitate the description of vectors linking any two points. For example, the vector linking Sydney and London has a distance of 16,994 km bearing 302.47°. This vector passes through cities such as Ambon, Manado, and Tacheng. Consequently, vectors have many practical applications, including GPS navigation. The trust ontology is similarly specified as a Cartesian coordinate system and hypothesized to facilitate the description of word vectors. As antonyms have previously been recognized as opposites on a vector passing through the origin, this research posited that word vectors should comprise multiple words laying on a vector, as shown in Figure 1. Using freezing-cold-hot-boiling as an example, freezing and cold are synonyms, hot and boiling are synonyms, freezing and boiling are antonyms, and cold and hot are antonyms. As the compass ontology is pragmatically divided into eight cardinal and intercardinal vectors, eight cardinal vectors were proposed to span the trust ontology, as shown in Figure 2.

### 1.4 Metrology

The purpose of a taxonomy is to aid the accurate and precise classification and measurement of phenomena (Bailey, 1994). Metrology is the science of accurate and precise measurement, of which the International System of Units is arguably the best-known example (Fanton, 2019). Metrology extends throughout the natural sciences, technology, industry, and commerce (Reichert, 2022). Metrology has a specific vocabulary for describing each element of the measurement process (BIPM, 2008). For example, the measurand is the quantity to be measured. Metrology also requires the specification of measurement units and the definition of the instrument to conduct the measurement. While measurement is a primary objective of social science, few social science measurement systems are metrologically compliant (Uher, 2020; Fisher and Stenner, 2023; Vieira and Monteiro, 2023); consequently, social science measurements are sometimes criticized as unreproducible (Wiggins and Christopherson, 2019). This research aimed to make the trust ontology and taxonomy metrologically compliant. Adopting metrological measurement offers the possibility of using the established methods of natural science to verify accuracy, precision, and validity in the social sciences.

### 1.5 Latent and manifest variables

Scientific models often combine manifest and latent variables. Manifest variables are observable and measurable. Conversely, latent variables are not directly observable, but their existence may be inferred if they facilitate the accurate prediction of manifest variables. For example, latent (unseen) magnetism is a widely accepted explanation for why compasses manifestly point North. The English language encompasses words with both latent and manifest meanings. For example, verbs describe manifest behaviors like running, singing, and dancing. Abstract nouns describe non-physical ideas or states like trust, distrust, happiness, sadness, love, and hate. These abstract nouns represent unseen psychological states that result in behaviors described by verbs. The psychological Attachment Theory combines manifest and latent variables (Ainsworth and Bowlby, 1991). The latent variables are view-of-self and view-of-other, sometimes renamed self-trust and other-trust (Parkes, 2013). Individuals with a positive view of self (self-trust) and a positive view of others (other-trust) are said to be securely attached. Ainsworth and Bowlby assert that secure attachment (a latent variable) manifests in prosocial behaviors like touching, smiling, and hugging (Barnett et al., 2022). The importance of latent variables to psychological theory has long been recognized (Markus and Borsboom, 2013). This research seeks to include both latent and manifest dimensional analysis formally. Therefore, in this research, the lexicon curation process aimed to include words congruent with the manifestations of dominance and affiliation and words congruent with the latent variables self-trust and other-trust.

### 1.6 Atlas

This research extends the Atlas lexicon and ontology to achieve a metrologically compliant taxonomy (Mobbs, 2020). The Atlas lexicon was initially developed to explore theories of personality and comprised over 20,000 nouns, adjectives, verbs, and idioms; however, the Atlas omitted gerunds and adverbs, and did not distinguish between nouns and abstract nouns. The original Atlas ontology was described in terms of the manifest dimensions of affiliation and dominance, but it did not propose corresponding latent dimensions (Mobbs, 2020). The Atlas’ dimensions of affiliation and dominance have been extensively investigated in social and emotional research. However, alternative descriptions have often been used for the affiliation dimension, such as warmth, friendliness, communion, and cooperation (Leary, 1957; Conte and Plutchik, 1981; Locke, 2014; van Kleef and Côté, 2022), and alternatives, such as agency and self-efficacy, have been used for the dominance dimension (Bandura, 1982; Abele and Wojciszke, 2018).

A deficit of the Atlas taxonomy is that it did not stipulate or identify the latent variables supporting the manifestations of affiliation and dominance. This research adopts Parkes’ description of Attachment Theory to overcome this deficit (Parkes, 2013). Therefore, this research hypothesizes that self-trust is the latent variable manifesting in dominant behaviors, and other-trust is the latent variable manifesting in cooperative behaviors. This research, therefore, endeavors to taxonomize the words congruent with the manifestations of dominant and cooperative behavior and words congruent with the latent dimensions of self-trust and other-trust within a single taxonomy. Therefore, some words in the lexicon will be more readily associated with dominance and cooperation, whereas others will be more readily associated with trust. The thesaurus provides synonyms and antonyms that link the latent variables of self-trust and other-trust with the manifestations of dominance and affiliation. A single taxonomy encompassing words relating to the manifestations of dominance and affiliation and the latent variables self-trust and other-trust constitutes evidence supporting the hypothesis.

This research continues with the Atlas ontology’s pragmatic division of the axes into five ordinal divisions, thus creating a square matrix of 25 cells. According to the Atlas ontology, words relating to personality, emotion, and behavior could be categorized into one of these 25 cells. The original Atlas taxonomization was performed manually using a Delphi process with a panel of experts (Hsu and Sandford, 2007). The present research supplanted the manual process with a machine-learning approach. It also expanded the Atlas lexicon to encompass words pertinent to all social sciences and the latent variables self-trust and other-trust.

## 2 Materials and methods

This research aims to curate a lexicon that forms the basis of a taxonomy. Once optimized, the objective is to identify features, such as symmetry and vectors, that are common in natural science theories. The sections below detail the methods used to curate the trust lexicon, extend and optimize the Atlas taxonomy, and identify vectors and symmetries.

### 2.1 Lexicon curation

The Atlas lexicon was extended in five ways to form the trust lexicon.

1. The root word was identified for each cataloged word using Python’s NLTK (Bird et al., 2009) stemming library and manual review. All words associated with that root word were identified using a dictionary and added to the lexicon (Merriam-Webster, 2021; Oxford University Press, 2022). For example, the word *cheerful* was recognized as a root word. Words derived from this root word not previously included in the lexicon were *cheered*, *cheerfully*, *cheerily*, and *cheering*. These four words were, therefore, added to the lexicon. A new column was included in the database to include the root words.
2. New words were identified by manually reviewing the synonyms and antonyms of words previously included in the lexicon (Merriam-Webster, 2021; Oxford University Press, 2022). Words with any information relevant to social interactions, dominance, affiliation, cooperation, self-trust, and other-trust were included. Focusing on synonyms and antonyms was pragmatically adopted to avoid reviewing all 300,000 English words. For example, the word lachrymose was not contained in the original lexicon. By manually examining the synonyms of sorrowful, lachrymose was identified as suitable for inclusion in the lexicon.
3. New words were identified by manually reviewing the freely available WordNet list of words (Miller, 1995). Words with any information relating to social interaction, dominance, affiliation, cooperation, self-trust, and other-trust were added to the lexicon. This process replicates the process adopted by Galton (1884), Allport and Odbert (1936), and Norman (1967), and others, where a sequential review of the dictionary was performed to identify suitable words that were not previously included in the lexicon.
4. A manual review of all words in the lexicon was conducted to identify prefixes and suffixes. For example, *disagreeable* has the prefix *dis* and the suffix *able*. New columns were included in the database to record prefixes and suffixes.
5. The part of speech was identified using the Oxford Dictionary classification (Oxford University Press, 2022). Where Oxford identified multiple parts of speech, the part of speech of the majority of synonyms and antonyms was adopted. The original Atlas lexicon recorded the parts of speech for all words in the lexicon. As new words were added to the lexicon, the part of speech was also recorded in the database.

### 2.2 Taxonomy optimization

**Figure 3:**
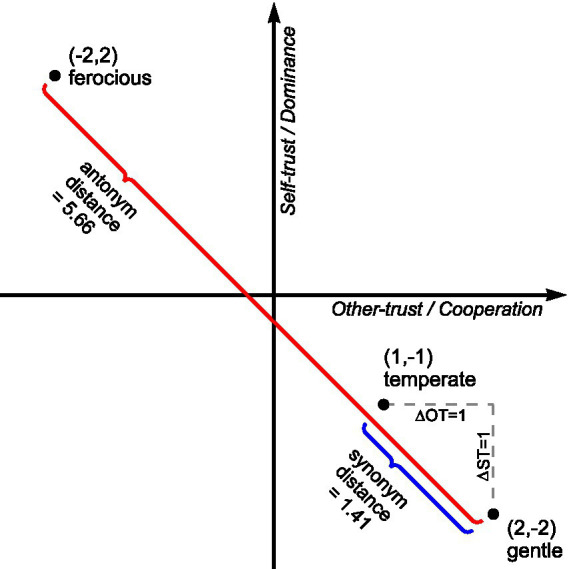
Taxonomy optimization. In this example, the location of three words, ferocious, temperate, and gentle, are shown on the Cartesian plane. ΔST is the difference in self-trust scores, and ΔOT is the difference in other-trust scores. The Pythagorean theorem is used to calculate the distance between synonyms and antonyms, in this instance, 1.41 and 5.66, respectively (see Equations 2, 3). An ideal taxonomy has similar phenomena that are tightly clustered and dissimilar phenomena that are widely separated. The taxonomy optimization process seeks to arrange the words on the Cartesian plane so that the average distance between antonyms is maximized and, concurrently, the average distance between synonyms is minimized.

Taxonomization is the empirical process of concurrently clustering similar phenomena by minimizing the distance between them and separating dissimilar phenomena (Bailey, 1994). Therefore, this research taxonomized the trust lexicon using a Gradient Descent approach to concurrently minimize the distance between synonyms and maximize the distance between antonyms. The unpaired t-score was employed as the objective function to measure taxonomic efficacy. The t-score was calculated by comparing the average distance between synonyms and the average distance between antonyms. For example, consider the synonyms gentle and temperate, initially located in cells (2,−2) and (1,−1), and ferocious, the antonym of gentle located in cell (−2,2) (Mobbs, 2020); see Figure 3. The Pythagorean theorem for right-angle triangles is used to calculate the distance between points on a Cartesian plane (see Equation 1), where ΔST is the difference in self-trust, and ΔOT is the difference in other-trust scores. Therefore, the geometric distance for synonyms is 1.41 (see Equation 2), and antonyms are 5.66 (see Equation 3). The t-score is calculated by first calculating the average distance and standard deviation for all synonym and antonym pairs. The calculation used the Python SciPy library (Virtanen et al., 2020). The *t*-score is maximized when the average distance between synonyms is minimized, and the average distance between antonyms is simultaneously maximized.


(1) $$Distance=\sqrt{\Delta ST^{2}+\Delta OT^{2}}$$

(2) $$Synonym\;Distance=\sqrt{{\left(2-1\right)}^{2}+{\left(-2-\left(-1\right)\right)}^{2}}=\sqrt{1^{2}+1^{2}}=1.41$$

(3) $$\begin{array}{l} Antonym\;Distance\\ =\sqrt{{\left(2-\left(-2\right)\right)}^{2}+{\left(-2-\left(-2\right)\right)}^{2}}=\sqrt{4^{2}+4^{2}}=5.66 \end{array}$$

Maximizing the taxonomic efficacy entailed iteratively trialing each word in each of the 25 cells of the trust taxonomy and calculating the t-score. The word’s location was altered to reflect the optimal t-score. This Gradient Descent optimization process was automated and repeated millions of times until improvements in the t-score were marginal (Ray, 2019). Manual intervention was required for a minority of words in which the synonyms are associated with divergent meanings. For example, the word *awful* has synonyms *horrible* and *fabulous*; however, the words *horrible* and *fabulous* are antonyms.

The Python code implemented two constraints to enhance processing efficiency and the identification of symmetry. First, the derivatives of root words were constrained to be located symmetrically, so for root words with coordinates (x, y), the derivative words were constrained to be at (x,y), (−x,y), (x,−y), or (−x,−y). Second, antonym reflection through the origin of the Cartesian plane was favorably weighted so that for words with coordinates (x,y), the antonym’s favored location was (−x,−y). The justification of these constraints is an empirical question. If the optimization results in an efficacious taxonomy, these constraints are supported. The Python code is available at doi.org/10.6084/m9.figshare.c.6918955.

### 2.3 Database design

A Google spreadsheet was selected to implement the lexicon and taxonomy database. Google Sheets was chosen because of the availability of Python Application Programming Interfaces (APIs), ease of user input for adding new words and columns such as prefixes and suffices, and rollback capability. The primary columns for the database were:

1. The Word (string).
2. The root word (string).
3. Self-trust score (integer).
4. Other-trust score (integer).
5. Part of speech (string).
6. Synonym count (integer).
7. Antonym count (integer).
8. Prefix (string).
9. Suffix (string).

A database copy is included as Supplementary Information in CSV and JSON formats. An additional database of synonyms and antonyms was maintained in JSON format. This second database contains the intellectual property of Oxford and Merriam-Webster. The Python program automatically populates this second database upon the license purchase. Python APIs were used to extract the data from the Google Sheets. The Python program automated all aspects of the thesaurus download and analysis process. However, the results of the optimization process required the manual update of the Google Sheet upon completing each iteration of the optimization process.

### 2.4 Vector identification

To identify word vectors, as depicted in Figure 1, the first step was to enumerate the set of all possible synonym pairs using Python. The Second step was to use Python to create the set of all four-word tuples where the inner pair were antonyms, and the outer pair were antonyms, where tuples are ordered sets of elements. From this set, the tuples were eliminated unless they conformed to the following:

1. The left-pair of words (positions 1 and 2) and right-pair of words (positions 3 and 4) in the tuple were synonyms.
2. The outer pair of words (positions 1 and 4) was found on the outer edge of the taxonomy, and the inner pair of words (positions 2 and 3) was found on the innermost segment of the taxonomy.

So far, this set of four-word tuples constitutes a set of candidate word vectors. However, some candidate word vectors were disconcordant. Therefore, the tuples were further improved by eliminating them unless they conformed to the following additional requirements:

1. The words must all share the same suffix, particularly for common suffixes such as *ed*, *ing*, and *ly*.
2. Words with prefixes indicating non-opposite antimony (Ding and Huang, 2013) were excluded, such as *un* and *non*. Words with opposite meanings were included, such as *dis* and *anti*.
3. The words must comprise the same part of speech, for example, nouns.

The Python code is available at doi.org/10.6084/m9.figshare.c.6918955.

## 3 Results

### 3.1 Lexicon curation

The 20,669-word Atlas lexicon was expanded to form a 30,072-word trust lexicon. A comparison between the Atlas and trust lexicons is shown in Table 1. The interaction between parts of speech and prefixes and suffixes is shown in Table 2. No pronouns, prepositions, conjunctions, or interjections were identified as suitable for inclusion in the lexicon. Nouns were divided into abstract nouns and nouns. Multiple-word idioms were removed from the lexicon due to having an indeterminate part of speech. The associations between suffixes and parts of speech were consistent with established rules of grammar. For example, over 90% of words with the suffixes *ism* and *ness* were abstract nouns, and over 99% of words with the suffix *ing* were gerunds. There were 8,072 root words; for example, the root word *belief* had 25 derivatives, including *believe*, *believer*, *non-belief*, and *disbelief*. The word *heart* had 72 derivatives, the most for any root word. For 2,254 root words, there were no derivatives other than the root word itself. The complete database of words is available at doi.org/10.6084/m9.figshare.c.6918955.

**Table 1:** Atlas and trust lexicon comparison.

Part of speech	Atlas lexicon (2020)	Trust lexicon (2023)
Adjectives	4,527	9,877
Nouns	6,432	3,297
Nouns–abstract	–	5,750
Verbs	3,124	4,682
Adverbs	–	1,543
Idioms	6,598	–
Total	20,669	30,072
Root words	5,958	8,072

**Table 2:** Word frequency analysis: suffix v part of speech.

Suffix	Abstract	Adjective	Adverb	Gerund	Noun	Verb	Grand total
(No suffix)	1,104	2,099	18		1,664	3,451	8,336
ing	6	87		4,923	20	1	5,037
ed	5	4,009			6	5	4,025
ness	2,015				4	2	2,021
ly		14	1,521		1		1,536
y	273	803	4		156	193	1,429
er	25	56			847	218	1,146
Other	2,322	2,809			599	812	6,542
Total	5,750	9,877	1,543	4,923	3,297	4,682	30,072

Approximately 400 words reflecting aspects of cognition, such as savant, awake, drowsy, delirious, psychosis, and comatose, were initially included in the lexicon curation but later excluded. These words were excluded because they were unrelated to the axes of self-trust, other-trust, dominance, or cooperation.

### 3.2 Optimization

The Python optimization process was iteratively run thousands of times, with each iteration taking a few hours to complete. After each iteration, the list of recommended word movements was manually evaluated. For example, a recommendation may be to move a particular word from (1,1) to (1,2). After each iteration, the word movements achieving the maximum individual increases in t-score were manually selected, and the database was manually updated. This process was repeated until the incremental improvements in *t*-score became miniscule. When the iterative process was completed, the t-score was −1,000.4. The *t*-score of −1,000 for the trust taxonomy was a substantial improvement over the Atlas taxonomy’s *t*-score of −427.3. For the 679,155 synonym pairs, the mode, mean, and standard deviations were 0, 1.16, and 1.26, respectively. For the 211,426 antonym pairs, the mode, mean, and standard deviations were 3.61, 3.66, and 0.93, respectively.

**Figure 4:**
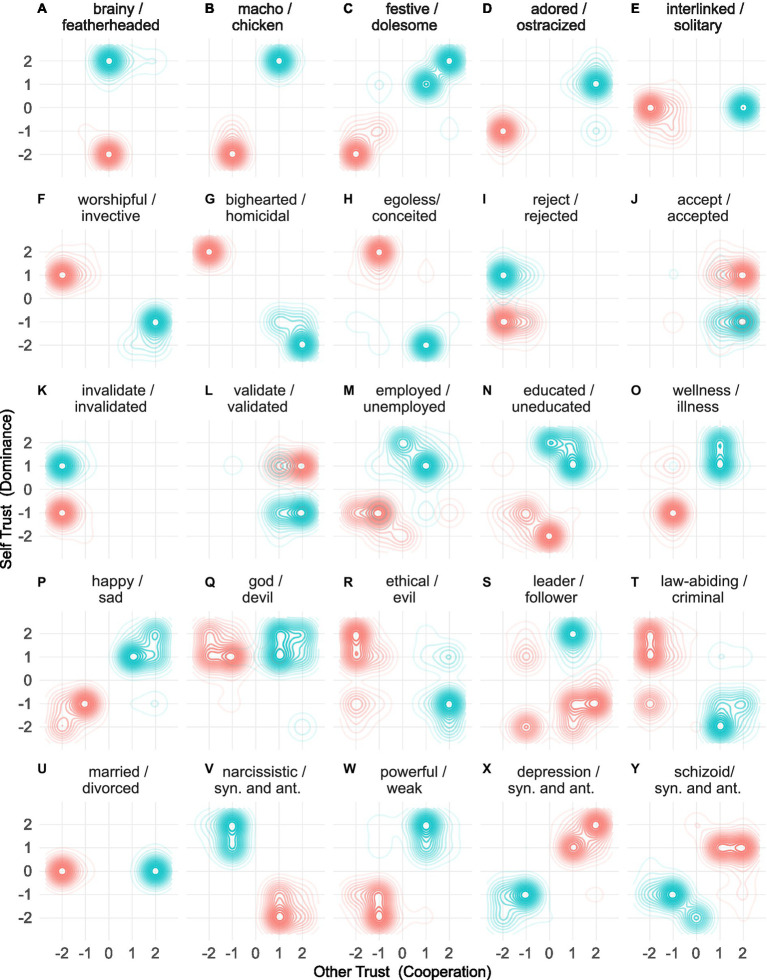
Synonym and antonym density plots. Density plots visually represent the location of synonyms and antonyms on the Cartesian plane. **(A–H)** Example density plots for 16 words located in the outer layer of the taxonomy. **(I–L)** Horizontal symmetry of verbs and adjectives with the suffix ed. **(M–Y)** Synonym density plots with words pertinent to the social sciences, law, economics, education, and psychology.

Because the trust ontology was defined as a Cartesian coordinate system, standard visualization tools such as density plots were used to visualize the taxonomy. Figures 4A–H shows density plots for 16 words, demonstrating the tight clustering of synonyms and wide separation of antonyms, reflecting the taxonomy’s efficacy. A visualization of all 30,000 words is available in the Supplementary Information available at doi.org/10.6084/m9.figshare.c.6918955. Of the 30,072 words in the lexicon, 23,292 had antonyms. Of these, 9,779 (42.0%) achieved perfect separation between the synonyms and antonyms with no overlap between the synonyms and antonyms. Words pertinent to several social sciences are visualized in Figures 4N–Y.

### 3.3 Symmetry

Most antonyms, 66.4%, were located on vectors passing through the origin. Of these, 38.8% were symmetrically reflected through the origin, and 27.6% were found elsewhere on the vector. For example, the antonyms *claim* and *disclaim* were located at (2,−1) and (−2,1). A minority of antonyms, 33.6%, were not found on the vector passing through the origin. For example, the word *well* is located at (1,1); therefore, its antonyms would be expected to be found in cell (−1,−1); however, the antonym *wounded* is in cell (−2,−1). The number of antonym pairs on a vector was 140,432 (66.4%). The one-tailed multinomial probability of 140,432 pairs being located on a vector is highly significant [Bin (*n* = 211,426, *k* = 140,432, *q* = 0.08) = 990(z), *p* = 0]. It was, therefore, inferred that the optimization process symmetrically arranged antonyms at opposite ends of vectors passing through the origin.

In addition to the symmetry of antonyms, two other forms of emergent symmetry were observed. There were 14,290 word pairs created from words with the same root and prefix. Of these pairs, 8,278 (57.9%) were colocated, an invariant symmetry, and 3,997 (28.0%) were horizontally symmetric, reflected across the horizontal axis. Of the 3,997 horizontally symmetric word pairs, 1,950 (48.8%) were word pairs comprising an adjective and verb. Of these, 1,345 (69.0%) comprised an adjective with the suffix *ed* and an unsuffixed verb. See Figures 4I–L. A qualitative review suggests that the word pair will exhibit horizontal symmetry if the verb relates to one party and the adjective to the other. For example, the horizontally symmetric word pair *reject* and *rejected* are located in cells (−2,2) and (−2,−2), respectively; see Figure 4I. The words are colocated, invariant symmetry, when the response is that of the person acting. Notably, words in the trust lexicon with the suffix *ed* were primarily adjectives rather than past participles. This is because the reference thesauri rarely include synonyms for past participles, but they do include synonyms and antonyms for adjectives.

In summary, three types of symmetry were observed (Glattfelder, 2019):

1. Reflection through the origin: Antonym pairs are reflected through the origin of the taxonomy’s Cartesian plane, mathematically represented as (x,y) → (−x,−y).
2. Horizontal symmetry: Verb/Adjective word pairs horizontally reflect across the horizontal axis of the taxonomy’s Cartesian plane, mathematically represented as (x,y) → (x,−y).
3. Invariant symmetry: Pairs are colocated in the same cell of the taxonomy, mathematically represented as (x,y) → (x,y).

### 3.4 Vectors

**Figure 5:**
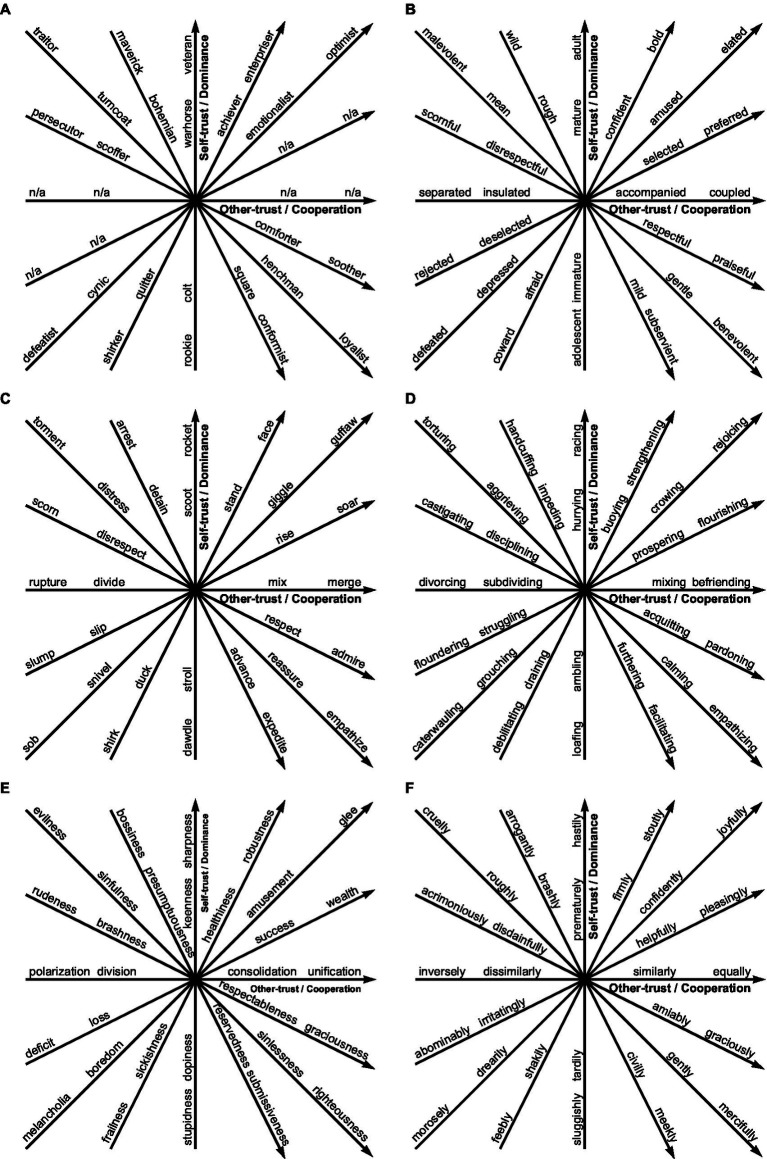
Example vectors spanning the taxonomy. **(A)** Noun **(B)** Adjectives **(C)** Verbs **(D)** Gerunds **(E)** Abstract Nouns **(F)** Adverbs. These vectors are consonant combinations of words related as synonyms and antonyms, created using the process as shown in Figure 2. **(A)** No noun vectors were identified for two of the eight cardinal vectors, denoted by n/a. Nouns generally had fewer synonyms than other parts of speech, and therefore, fewer noun vectors were identified compared to other parts of speech.

The vector identification process yielded 226,250 vectors, as shown in Figure 5. Many words were duplicated in these vectors; for example, the word *poking* appeared in 6,678 (3.0%) of the 226,250 vectors. The 226,250 vectors were reduced to 942 using 3,768 unique words. The number of vectors was further reduced by removing antiquated and rarely used words, resulting in 573 vectors that use 2,292 unique modern and familiar words (Mandera et al., 2020). No noun vectors were identified for two of the eight cardinal vectors; see Figure 5A. These vectors have been denoted with n/a. Nouns generally had fewer synonyms than other parts of speech, and therefore, fewer noun vectors were identified compared to the non-noun parts of speech. The complete database of vectors is available at doi.org/10.6084/m9.figshare.c.6918955.

## 4 Discussion

This research hypothesized that self-trust and other-trust are the latent variables manifesting in dominant and cooperative behaviors. Words reflecting these concepts were included in a single lexicon to test this hypothesis. For example, *believable* is an adjectival descriptor of an individual’s personality, presumably because the individual is trustworthy and warrants other-trust manifesting in cooperative behaviors. *Believe* is the corresponding verb for *believable*, and *belief* is the abstract noun indicating other-trust. Correspondingly, *disbelief* indicates negative other-trust. Therefore, the four words *belief*, *disbelief*, *believable*, and *believe* should all be included in the lexicon. The optimization result of this analysis (*t* = 1,000) is evidence that other-trust, self-trust, dominance, and cooperation can reasonably be contained in a single lexicon, and the strength of associations between other-trust and cooperation and self-trust and dominance have been empirically well established (Leary, 1957; Conte and Plutchik, 1981; Locke, 2014; Abele and Wojciszke, 2018; Van Kleef and Côté, 2022). This analysis confirms the strength of linguistic associations, supporting the hypothesis that self-trust and other-trust are latent variables manifesting in dominant and cooperative behaviors.

This research curated a comprehensive lexicon spanning all social sciences. The ontology led to a taxonomy with an exceptional t-score, suggesting that the dimensions of the ontology, self-trust and other-trust, manifesting in dominant and cooperative behaviors, are fundamental to human social existence. The implications of these dimensions still need to be explored. Figures 4I–R demonstrate that concepts of profound human importance over many centuries, such as religion, ethics, leadership, power, and legal concepts, can now be visualized, differentiated, and metrologically measured and compared using the trust taxonomy.

This research built on prior claims that trust is foundational to all social science domains to develop a trust ontology and taxonomy (Hosking, 2014; Dunning et al., 2019; Weiss et al., 2021). Extending a taxonomy of personality psychology previously developed by domain experts (Mobbs, 2020), this research used a machine-learning approach to optimize the taxonomy. A t-score of this magnitude confirms the efficacy of the taxonomy, as similar phenomena (synonyms) are tightly clustered, and dissimilar phenomena (antonyms) are widely separated. The trust taxonomy confirms the established symmetry of antonyms, defined as points on a vector equidistant from an origin. This research further identified a horizontal symmetry associated with the suffix *ed*. This emergent symmetry suggests that the interactions within dyads may be predictable. For example, the verb *reject* and adjective *rejected* are horizontally reflected, located at (−2,2) and (−2,−2), respectively, as shown in Figure 4I. The emergence of symmetry and vectors implies a level of realism unobserved in previous taxonomies of social science.

This research suggests that words in the trust lexicon and cartographic placenames serve a corresponding purpose. As humans agree on placenames for points of interest, the placename’s location is specified using Cartesian coordinates with axes of latitude and longitude. Similarly, humans develop words representing concepts of interest; the 30,000 words in the trust lexicon can be specified using Cartesian coordinates with axes self-trust and other-trust. By convention, latitude and longitude are pragmatically divided into 360 equidistant divisions known as degrees. Similarly, the trust ontology pragmatically divided self-trust and other-trust into five equidistant divisions. Therefore, in the same way that the vector (distance and direction) between two placenames can be calculated, the vector between words in the trust lexicon can be calculated. Historically, several social sciences have typically measured the differences between phenomena using correlations. However, correlations do not facilitate the measurement of distance between phenomena. The benefit of a Cartesian ontology and taxonomy is that it may enable measurement of the difference between phenomena in terms of direction and angle, similar to GPS navigation. For example, individuals may need to increase both self-trust and other-trust to move from sadness to happiness, see Figure 4P. Future clinical research may investigate efficacious therapies for developing self-trust and other-trust.

The inclusion of all parts of speech in the trust taxonomy may help identify behaviors (verbs) typically associated with personality traits (adjectives) and emotions (abstract nouns). For example, the taxonomy locates the abstract noun *love* at (2,−2). Examples of verbs colocated with *love* include *pamper*, *protect*, and *swaddle*. Notably, *pamper*, *protect*, and *swaddle* are not synonyms of *love* identified in the source thesauri (Merriam-Webster, 2021; Oxford University Press, 2022). Similarly, the trust taxonomy locates the adjective *abusive* in cell (−2,2), with colocated verbs including *assault*, *injure*, and *torment*. Again, these words are not presently recognized as synonyms for *abusive* in the source thesauri. These are examples of how the trust taxonomy could identify new word associations not previously recognized in commercial thesauri. However, this research did not investigate the type of interaction between colocated words. Future research may investigate the strength of correlations between such words and whether the interaction indicates causality.

These findings also have practical implications. Generating word vectors with commercially curated synonyms and antonyms is a pragmatic approach, resulting in a consonant spectrum of words with conceptually similar meanings. As all vectors start and end at the extremities of the taxonomy and intersect the geometric origin (0,0), it ensures that the spectrum identifies maximally opposite endpoints and sequentially ordered intermediate segments on the spectrum, see Figure 1. These spectrums may assist psychometric testing by augmenting the use of Likert-type scales. For example, “*I know how to captivate people*” is a common question in personality questionnaires (DeYoung et al., 2007). Respondents are invited to score on a scale of how accurately this statement describes them. One criticism of the Likert scale approach is that such questions are subjective and not quantitatively comparable between respondents (Boag, 2015). Conversely, with the current approach, the concept of captivation is found in 15 vectors, of which repelling-boring-entertaining-captivating is an example. The Likert-type question could be augmented with a question inviting the respondent to select the word on the vector that best describes them.

At the same time, the trust taxonomy was developed to be metrologically compliant to make social science measurements similar to measurements in the natural sciences (BIPM, 2008). The trust ontology specifies the measurands, self-trust and other-trust, and the five divisions of self-trust and other-trust serve as the units of measurement. The trust taxonomy is the instrument by which the measurands are measured. The metrologically compliant trust taxonomy offers the possibility of a new approach to measurement in and across the social sciences. This new approach replaces Likert-type scales (Jebb et al., 2021) with the direct measurement of trust using English words. Adopting metrological measurement offers the possibility of using the established methods of natural science to verify accuracy, precision, and validity in the social sciences.

Despite trust’s recognized foundational importance to social science, few theories have explicitly identified trust as an endogenous or exogenous variable. The proposed trust ontology provides a basis for understanding language’s endogenous content and exogenous implications. To the extent that existing social science theories are described with words, these theories may now be explicitly related to trust. For example, depression is a symptom of some personality disorders. The trust ontology suggests the reinterpretation of depression as having negative other-trust (distrusting others) and negative self-trust (distrusting self). Conversely, happiness indicates the state of being trustful of oneself and others. These interpretations suggested by the trust taxonomy, derived using commercial thesauri, could be the subject of future empirical research to confirm such interpretations.

### 4.1 Limitations and future directions

There are several limitations of this research and future research opportunities. The primary limitation is that the analysis exclusively used English, and the taxonomy must be demonstrated to be extensible to other languages. The pragmatic selection of a five-by-five taxonomy could be empirically compared with taxonomies with increased measurement resolution. Furthermore, the achieved efficacy of the trust ontology and emergent vectors and symmetries do not imply that this taxonomy is maximally efficacious, complete, or realistic in all respects. It is also possible that words are in unexpected locations. The trust ontology and taxonomy are expected to improve in several ways, such as through expert review or new computational approaches. Trust could also be replaced with an alternate concept that achieves superior taxonomic efficacy for subsets of the lexicon or for the lexicon as a whole. The methods developed in this research can empirically assist in furthering these investigations.

As with all theory development, substantial empirical research must assess the theory’s realism and utility. Such empirical investigations could assess the associations between words in the same matrix cells where the words have not previously been recognized as synonyms. For example, the words *fit* and *personable* are not listed as synonyms in the source thesauri, yet they are placed in the same matrix cell. Empirically establishing correlations between such concepts would help support the ontology’s and taxonomy’s realism and utility. Future research must also assess the variance between the lexical and semantic meanings of words in the Trust lexicon. The lexical approach in this analysis infers meaning from the synonyms and antonyms used in thesauri. However, individuals may interpret these words along a spectrum of meaning. How these spectrums of meaning may impact the trust ontology and taxonomy usage has yet to be assessed.

This research was founded on prior research that identified the significance of trust to each social science (Hosking, 2014; Dunning et al., 2019; Krueger and Meyer-Lindenberg, 2019; Schilke et al., 2021; Weiss et al., 2021). Future empirical research must confirm that the Trust ontology and taxonomy are relevant to each social science.

Geographic placenames are accurately specified with longitude and latitude; however, some placenames communicate additional information. For example, Mount Everest is universally understood to mean the Earth’s highest peak, in addition to specifying its latitude and longitude. Similarly, some words in the trust lexicon may communicate additional information, such as aspects of power relevant to political science (Lowande and Rogowski, 2021), intelligence pertinent to education (Anglim et al., 2022), or cognition (see Section 3.1). The trust lexicon included all words with any level of trust information. Future research may use the ontology-lexicon-taxonomy approach developed in this research to identify homogeneous sub-lexicons that may have alternate or additional dimensionality. These newly identified dimensions may be orthogonal to the concepts of trust or more fundamental cognitive processes from which trust emerges.

This research was predicated on the hypothesis that self-trust causes dominant behaviors and other-trust causes cooperative behaviors. This research has demonstrated the linguistic associations between other-trust and cooperation and self-trust and dominance. However, this research does not establish the direction of causality. Therefore, the direction of causality may be in the reverse direction; that is, cooperative behaviors cause other-trust. Alternatively, causation may be reciprocal; that is, cooperation causes other-trust, and equally, other-trust causes cooperation. The reciprocal nature of trust and cooperation has been identified in organizational contexts (Grossman and Feitosa, 2018).

The trust taxonomy includes words technically defined in various social science domains. For example, the word *criminal* (see Figure 4T) is defined by legislation in each jurisdiction, and words such as *narcissistic*, *depression,* and *schizoid* (see Figures 4V,X,Y) have clinical meanings (American Psychiatric Association, 2013), and religions uniquely define their *god(s)* and *devil(s)* (see Figure 4Q). Future research must compare the linguistic meaning of these words, supported by synonym and antonym associations, in the context of their technical and cultural meanings. Future research must assess whether the ontology aligns with social behavior interpreted within these technical and cultural frameworks.

## 5 Conclusion

This research sought to conceptualize and test a unifying ontology of social science analogous to how quantum mechanics and general relativity unify the natural sciences. By utilizing the lexical hypothesis and machine learning methods, this research found the two-dimensional taxonomy of trust to have high levels of efficacy and realism, meaning that all social sciences share a common conceptual framework of self-trust and other-trust. The metrologically compliant measurement basis of the trust taxonomy and its identified word vectors and horizontal symmetry provides the social sciences with a measurement system that, with further development, can be as accurate and precise as the measurement systems used in the natural sciences. The present research breaks down barriers between intellectual disciplines by providing a common ontology across social sciences and a measurement system commensurate with the natural sciences, thus fostering a more comprehensive understanding of human experience.

## Data Availability

The datasets presented in this study can be found in online repositories. The names of the repository/repositories and accession number(s) can be found at: doi.org/10.6084/m9.figshare.c.6918955.
